# Exploring the impact of taurine on the biochemical properties of urate oxidase: response surface methodology and molecular dynamics simulation

**DOI:** 10.1186/s13036-023-00397-x

**Published:** 2024-01-22

**Authors:** Parisa Shahmoradipour, Maryam Zaboli, Masoud Torkzadeh-Mahani

**Affiliations:** 1 Department of Biotechnology, , Institute of Science, High Technology and Environmental Sciences, Graduate University of Advanced Technology, Kerman, Iran; 2 Department of chemistry, faculty of science, University of Birjand, Birjand, Iran

**Keywords:** Taurine, Molecular dynamics simulation, Urate oxidase, Response surface methodology, Molecular docking

## Abstract

This paper investigates the impact of taurine as an additive on the structural and functional stability of urate oxidase. First, the effect of the processing parameters for the stabilization of Urate Oxidase (UOX) using taurine was examined using the response surface methodology (RSM) and the central composite design (CCD) model. Also, the study examines thermodynamic and kinetic parameters as well as structural changes of urate oxidase with and without taurine. Fluorescence intensity changes indicated static quenching during taurine binding. The obtained result indicates that taurine has the ability to preserve the native structural conformation of UOX. Furthermore, molecular dynamics simulation is conducted in order to get insights into the alterations in the structure of urate oxidase in the absence and presence of taurine under optimal conditions. The molecular dynamics simulation section investigated the formation of hydrogen bonds (H-bonds) between different components as well as analysis of root mean square deviation (RMSD), root mean square fluctuations (RMSF) and secondary structure. Lower Cα-RMSD and RMSF values indicate greater stabilization of the taurine-treated UOX structure compared to the free enzyme. The results of molecular docking indicate that the binding of taurine to the UOX enzyme through hydrophobic interactions is associated with a negative value for the Gibbs free energy.

**Supplementary Information:**

The online version contains supplementary material available at 10.1186/s13036-023-00397-x.

## Introduction

Urate oxidase (UOX) (EC 1.7.3.3), is an enzyme that binds to copper and catalyzes the chemical reaction of uric acid oxidation, resulting in the production of hydrogen peroxide (H_2_O_2_) and 5-hydroxyisourate. Uricase has the potential to serve as a therapeutic enzyme for the purpose of reducing the concentration of uric acid in the plasma [1]. In marine animals, it is metabolized further to urea and glyoxylic acid and is thus not excreted [2–4]. Urate oxidase is frequently observed in bacterial and fungal organisms. Nevertheless, it is important to note that humans lack uricase enzymes as a result of genetic abnormalities in the urate oxidase gene [5, 6]. The utilization of recombinant uricase enzyme has proven to be effective in the management of hyperuricemia, exhibiting a minimal occurrence of hypersensitivity reactions and tophaceous gout [6–8]. Furthermore, the uricase enzyme serves as a reagent in clinical diagnostic kits utilized for the enzymatic quantification of uric acid [6, 7]. One of the significant challenges associated with the utilization of therapeutic enzymes is to their limited stability. One approach to improve protein stability is the utilization of additives [9]. Living organisms, including both prokaryotic and eukaryotic cells, when exposed to adverse environmental conditions, such as high salt concentrations, extreme temperatures, or low temperatures, employ a similar mechanism to safeguard their proteins. This mechanism involves the synthesis of organic compounds known as osmolytes. Stabilizing osmolytes are organic compounds with a low molecular mass that raise the midpoint of thermal denaturation. They are divided into amino acids and their derivatives, polyhydric alcohols, sugars, and methylammonium derivatives [10–12]. Osmolytes can be classified into two distinct classes, namely compatible and counteracting, based on their functional mechanisms in relation to protein activity [13]. The mechanistic effect of each osmolyte on the performance and stability of the protein is unique and special. Note that sometimes the proteins that have been exposed to osmolytes and folded are more active than in their natural state [13, 14]. Taurine osmolyte is used to stabilize the structure of proteins. Taurine osmolyte is an organic acid with a molar mass of 125.14 g/mol found in many natural food sources. Taurine osmolyte is derived from cysteine and biosynthesized in the body; it is also produced via chemical synthesis for commercial purposes. Proteins that have high thermal stability have many applications in the field of biotechnology. The subject discussed, which is of great importance, is increasing the resistance of proteins in acute conditions. The comprehensive assessment of the stability and functionality of the uricase enzyme in the presence of osmolytes has yet to be fully elucidated. In this study, the correlation between protein stability and activity alterations when exposed to taurine osmolyte was examined. This study also looked at how taurine osmolyte affects the activity and structural stability of the recombinant urate oxidase enzyme. The investigation focused on assessing the impact of taurine on the enzymatic activity of recombinant uricase through the utilization of the response surface methodology (RSM) in order to determine the best conditions. The response surface method was used to achieve recommended optimal conditions, including the concentration of taurine osmolyte, incubation time, and temperature. Thermodynamic and kinetic parameters, and structural studies including intrinsic, and ANS fluorescence of pristine urate oxidase and taurine-treated uricase enzyme were examined. The obtained results indicated that the thermal stability of the untreated recombinant uricase enzyme was comparatively poorer in comparison to the uricase enzyme treated with taurine osmolyte. The study employed atomistic molecular dynamics (MD) simulations to get structural insights into the experimental findings and to offer further information regarding the impact of taurine. Also, MD simulation was applied to check the stability and flexibility of the formed complexes [15]. The findings indicate that the presence of taurine led to an increase in the proportion of α-helical and β-sheet structures in the recombination uricase enzyme.

## Materials and methods

### Material

Boric acid (CAS No. 10043-35-3), Taurine (CAS No. 107-35-7; purity ≥ 99%), and uric acid (CAS No. 69-93-2, ≥ 99%) were from Sigma-Aldrich. The compounds KH2PO4 (CAS No. 7778-77-0) and K2HPO4 (CAS No. 11-4-7758) were procured from the Merck company. Affinity chromatography column of Ni-NTA resin for His6-tagged proteins was purchased from Qiagen. NaCl, yeast extract, Tris-HCl (CAS No. 1185-53-1), tryptone (CAS No. 91079-40-2). The agar medium was used for both liquid and solid culturing purposes. Bradford (Bio-Rad Protein) solution for the quantification of protein concentration, agarose, Isopropyl β-D-1-thiogalactopyranoside (IPTG) (CAS No. 367-93-1), sodium acetate, kanamycin antibiotic, and imidazole were obtained from Sina-clon Co, Iran. The plasmid extraction kit was purchased from Takara company, Japan. Acrylamide (CAS No. 79-06-1), sodium chloride (CAS No. 7647-14-5), glycine, and all SDS-PAGE chemicals were obtained from Merck company.

#### Microorganisms and media composition

The urate oxidase gene was expressed using the pET method developed by Novagen. The host organism for plasmid clones in this study was *Escherichia coli*, commonly referred to as *E. coli* (Invitrogen, Carlsbad, CA, USA). The strain of *E. coli* used in this study for protein expression is BL21 (DE3). The plasmid pET-28a was employed in this study to express the recombinant uricase enzyme. The expression was regulated by the T7-promoter, which was induced by isopropyl-β-D-1-thiogalactopyranoside (IPTG) obtained from Sigma Aldrich, USA. The bacterial strains were cultured using Luria-Bertani (LB) medium, which consisted of 1% (w/v) tryptone, 0.5% (w/v) yeast extract, and 1% (w/v) NaCl. For LB agar, 2% (w/v) agar was also used in the medium. The pH of the medium was adjusted to 7, and kanamycin was added at a concentration of 100 µg/ml as required.

#### Purification of the urate oxidase enzyme expressed in *E. Coli*

For recombinant uricase enzyme synthesis, the plasmid vector pET-28a was used as an expression vector, and the *Escherichia coli* BL21 strain was employed as the expression host. The cells obtained from a 100 ml culture were subjected to lysis and afterwards resuspended in 1–3 ml of Tris-HCL buffer solution (pH 8) with a concentration of 50 mmol. The process of sonication was performed on ice for a duration of 3 min, with a cycle of 70 and intervals of 0.5 s. The lysate was subjected to centrifugation at a speed of 13,000 g, at a temperature of 4 ˚C, for a duration of 20 min. The resulting supernatants after centrifuging were collected for the purpose of purification. The liquid portion of the sample (supernatant) was loaded onto chromatography columns containing Ni-Sepharose, and subsequent purification steps were carried out. Initially, the columns were washed using a wash solution with a concentration of 50 mM (pH 8). This wash buffer consisted of 2.5 M NaCl, 200 mM NaH_2_PO_4_, and 20 mM imidazole. The purpose of this washing step was to remove any nonspecific protein bonds that may have occurred within the columns. Following this, purification was performed utilizing an elution buffer comprising NaCl and NaH_2_PO_4_, along with different concentrations of imidazole ranging from 50 to 300 mM [16]. The protein concentration was determined using the Bradford test (Bio-Rad Protein, 1976), with bovine serum albumin (BSA) serving as the standard protein. In the present investigation, the SDS-PAGE technique, utilizing Laemmli’s buffer system, was utilized to determine the purity of the purified enzyme.

#### Uricase assay

The blank reaction mixture is composed of a Tris-HCl buffer with a concentration of 0.1 M and a pH of 9.5. Additionally, the mixture contains 1 mM of EDTA. The reaction mixture comprised 0.9 ml of air-saturated 0.1 M Tris–HCl buffer (pH 9.5) containing 1 millimolar EDTA, 0.1 millimolar uric acid, and 20 µl of urate oxidase enzyme solution. The activity of the uricase enzyme was assessed by measuring the decrease in absorbance at a wavelength of 293 nm. The one unit of enzyme activity (U) was defined by determining the amount of enzyme necessary to catalyze the conversion of 1 µmol of uric acid to allantoin within a duration of 1 min (definition A) under room temperature and pH 9.5 conditions [17]. The following equation can express the activity of recombinant uricase enzyme:

$${enzyme}_{\left(\raisebox{1ex}{unite}\!\left/ \!\raisebox{-1ex}{ml}\right.\right)}=\frac{({\varDelta A}_{293 nm}/minTest-{\varDelta A}_{293 nm}/minBlank)\left(\text{t}\text{o}\text{t}\text{a}\text{l}\,\text{v}\text{o}\text{l}\text{u}\text{m}\text{e}\right)\left(\text{d}\text{f}\right)}{\left(12.6\right) 0.02}$$

The symbol “df” represents the dilution factor, the value 12.6 corresponds to the millimolar (mM) extinction coefficient of uric acid at a wavelength of 293 nm, and the quantity 0.02 (ml) represents the volume of the uricase enzyme.

### RSM methodology

The influence of the variables affecting the activity of the urate oxidase enzyme in the presence of taurine osmolyte was investigated with a full fraction design. Three operating variables including temperatures, incubation time, and the concentration of taurine were examined. In order to optimize the three most prominent factors, 18 full factorial central composite designs were implemented using the response surface methodology. This was accomplished through the utilization of Design-Expert software version 11.

#### Optimum temperature, incubation time, and taurine concentration by RSM

In order to optimize the stability process of the uricase enzyme, central composite design (CCD) and the response surface methodology (RSM) were employed. This approach aimed to attain the utmost efficacy and maximum level of stability for the urate oxidase enzyme. Based on the first experimental findings, three factors were identified as the independent variables: incubation time (x1), taurine concentration (x2), and temperatures (x3). The response addressed in this study was the activity of the urate oxidase enzyme after stabilization, measured in units per milliliter (U/ml). The investigation focused on determining the optimal estimated temperature for the activity of the recombinant uricase enzyme. This was achieved by assessing the enzyme’s activity subsequent to pre-incubation at different temperatures, spanning from 16 to 40 ˚C, with intervals of 5 ˚C, for a duration of 6 min. In order to calculate the duration of the optimum incubation, the urate oxidase enzyme and taurine were subjected to incubation for a range of 5 to 35 min. The present study aimed to investigate and compare the catalytic activity of uricase enzymes in the presence and absence of taurine osmolyte under different temperature conditions and incubation times. To assess the impact of the optimal taurine concentration on the thermal stability of the recombinant urate oxidase enzyme, several concentrations of taurine osmolyte (0, 50, 150, 250, 350, and 450 mM) were examined with regard to the activity of the urate oxidase enzyme.

### Thermal inactivation

In this study, two sets of experiments were conducted to investigate the thermostability of the urate oxidase enzyme treated with taurine. In order to investigate the process of thermal inactivation, the recombinant uricase enzyme was subjected to various temperatures, specifically 40, 55, and 60 ˚C, for a duration of 1 h. During this incubation period, measurements were taken at 5-minute intervals. It is important to note that the uricase enzyme was incubated without the presence of its substrate, uric acid. Subsequently, the samples were subjected to a cooling process after being placed on ice for a duration ranging from 30 to 60 min, with the aim of reinstating their native folding structure. Then, the residual activities of the drawn enzyme samples were measured. The residual activities of the drawn enzyme samples vs time plots were employed to determine the melting temperature of the enzyme. The melting temperature is defined as the temperature at which half of the native enzyme molecules have been denatured to an inactive conformation. The experiment was continued until no further decrease in enzyme activity (A/A0) was observed, indicating that the enzyme had reached its minimal level of activity, A_min_. The A_min_ value refers to the minimal level of activity exhibited by an enzyme at a specific temperature, indicating the enzyme’s equilibrium condition and representing the overall quantity of active enzyme after cooling [18–20]. The experiment was conducted in triplicate, with each trial consisting of three measurements. The result presented is the average of these triple measurements.

### Kinetic parameters

A more comprehensive comprehension of the stability of the recombinant urate oxidase enzyme through the utilization of taurine osmolyte can be achieved by evaluating the kinetic parameters, namely the maximum reaction velocity (V_max_) and the Michaelis Menten constant (K_m_). In order to determine the Michaelis-Menten constant (K_m_) and maximum reaction rate (V_max_) of uricase, experimental solutions were prepared by combining 20 mmol of boric acid with varying amounts of uric acid as the substrate. The determination of the Michaelis-Menten constant and maximum reaction velocity values was conducted using the Lineweaver-Burk plot, as described by Eqs. 8 and 9, respectively [21]. The turnover number value (K_cat_) was obtained by dividing the maximum reaction velocity value by the final molar concentration of the recombinant urate oxidase enzyme. The experimental measurements for the untreated uricase enzyme and the uricase enzyme treated with taurine osmolyte were each replicated three times.

$${V}_{0}=\frac{{V}_{max}\left[S\right]}{{K}_{m}+\left[S\right]}$$

$$\frac{1}{{V}_{0}}=\frac{{K}_{m}}{{V}_{max}\left[S\right]}+\frac{1}{{V}_{max}}$$

In the Lineweaver-Burk equation, $\frac{{k}_{m}}{{V}_{max}}$ is the slope and $\frac{1}{{V}_{max}}$ is the y-intercept

### Fluorescence measurements

#### Intrinsic fluorescence

The experiment commenced by employing Amicon Ultra Centrifugal Filter Units to eliminate imidazole present in solutions containing purified uricase enzyme. Subsequently, desalting protein solutions were utilized to carry out the subsequent stages of the experiment. A Cary Eclipse fluorescence spectrophotometer (Varian) was used to assess the steady-state fluorescence of recombinant urate oxidase in the presence and absence of taurine osmolyte. The fluorescence spectra were obtained by exposing a sample to an excitation wavelength of 290 nm and measuring the emission wavelengths between 300 and 400 nm. This was done under varying concentrations of taurine at temperatures of 40, 55, and 60 °C. The temperature within the quartz cells was regulated using a thermostatically controlled circulating-water pump. The experiment involved the addition of the recombinant uricase enzyme at a concentration of 0.5 mg/mL to a Tris-HCl buffer solution with a volume of 170 mL. This experimental procedure was conducted over a concentration range spanning from 0 to 750 mM of taurine [22].

#### ANS fluorescence

The compound known as 8-anilino-1-naphthalenesulfonic acid (ANS) is a commonly employed fluorescent probe in the field of protein research, specifically for the purpose of characterizing protein binding sites. Initially, stock solutions of 8-anilino-1-naphthalenesulfonic acid (ANS) were produced using phosphate-buffered saline (PBS). Subsequently, the interactions between ANS and both treated and untreated enzymes were observed using a Cary Eclipse fluorescence spectrophotometer. All measurements were made at a temperature of 28 °C in the cuvette. Following the addition of ANS to the enzyme solution at a ratio of 1:30, the samples were thoroughly mixed and subsequently incubated in a light-restricted environment for a duration of 10 min before fluorescence measurements were taken. The excitation wavelength utilized for ANS fluorescence spectra was held constant at 370 nm, while emission wavelength scans were conducted within the range of 400 to 600 nm [23, 24].

### Computational methods

#### Molecular dynamics simulation

The MD simulations were done on the A. flavus UOX crystallographic structure resolved at 2.30 Å (PDB ID: 1R56 from Protein Data Bank) [25]. Recent structures indicate that the MD simulation can help describe the stabilization or destabilization of the protein structure in the presence of osmolyte [9, 26–29]. The MD calculations were done on the native and taurine-treated enzyme using the GROMACS software under the optimum conditions (4.50 M taurine and 28 °C) obtained from the RSM method. The velocity rescale thermostat and Berendsen algorithm were used to maintain the temperature and pressure of the system (300 K and 1 bar, respectively) [30, 31]. The particle-mesh Ewald (PME) algorithm was used for long-range electrostatics [32]. Van der Waal interactions were modeled using Lennard-Jones 6–12 potentials with 14 Å cut-off. Bond lengths were constrained using the Linear Constraint Solver (LINCS) algorithm [33]. The system was energy minimized using the steepest descent; after an equilibration of 500 ps at constant pressure and temperature, the system was subjected to 50 ns of molecular dynamics (MD). Trajectories were saved at 2 ps intervals, and trajectory analyses were performed using the programs of GROMACS suite. The leap-frog algorithm was used as an integrator with a 2 fs time step to integrate Newton’s equations of motion. The gmx hbond utility in the GROMACS was applied to measure the number of hydrogen bonds (H-bonds). Analysis of the H-bonds employed a donor-acceptor cutoff distance of 3.5 Å and acceptor-donor-hydrogen cutoff angle of 30°. The root mean square deviation (RMSD) was calculated by comparing the simulated protein structure during simulation with the reference structure [34]. This study applied Cα-RMSD values to estimate protein structure similarity. To assess the residual mobility of the protein, the root-mean-square fluctuation (RMSF) was calculated for the Cα atoms of each residue. Therefore, the RMSF of residues indicates the flexibility or rigidity regions within the protein structure where those residues are located [35], a higher RMSF value suggests lower stability [36]. The solvent-accessible surface area (SASA) was calculated, which indicates the solvent-accessible surface of protein residues. An increase or decrease in the SASA values demonstrates the changes in the exposed amino acid residues and could influence the protein tertiary structure. The SASA values were calculated for the 50 ns trajectory of molecular dynamics simulation for free and treated-UOX enzymes. Also, the secondary structure analysis was done with the DSSP program [37]. In this protocol, hydrogen bonding and the other geometrical features were used to determine the secondary structure of the enzyme. To ensure the reproducibility of our results, we conducted the MD simulation three times with varying initial structures. The results of three runs showed a high degree of similarity (the results can be seen in the electronic Supplementary Material). This work presents the average results obtained from three simulations.

#### Molecular docking

We used MGLTools 1.5.6 AutoDockTools (ADTs) along with AutoGrid 4.2 and AutoDock 4.2 to prepare and perform molecular docking calculations between UOX enzyme and taurine molecule. The Lamarckian Genetic Algorithm (LGA) [38] was utilized to perform docking calculations. The target protein file was prepared using AutoDock 4.2 tool by excluding heteroatoms, structural water molecules, and co-factors, while retaining the protein-associated residues. This involved several steps such as assigning AD4 type atoms, calculating Gasteiger charges for each atom in the macromolecule, and adding polar hydrogens. Adding polar hydrogens is crucial to ensure accurate calculation of partial charge, while keeping all other values as default. The LIGPLOT program was utilized to illustrate the strengths of intermolecular interactions, including hydrogen bonds and hydrophobic interactions [39]. The energy evaluations were set to 2,500,000 and 27,000 generations, with a population size of 150. The gene mutation rate was set at 0.02, while the gene crossover rate was set at 0.8.

## Results and discussion

### Experimental results

#### Protein purification

**Fig. 1:**
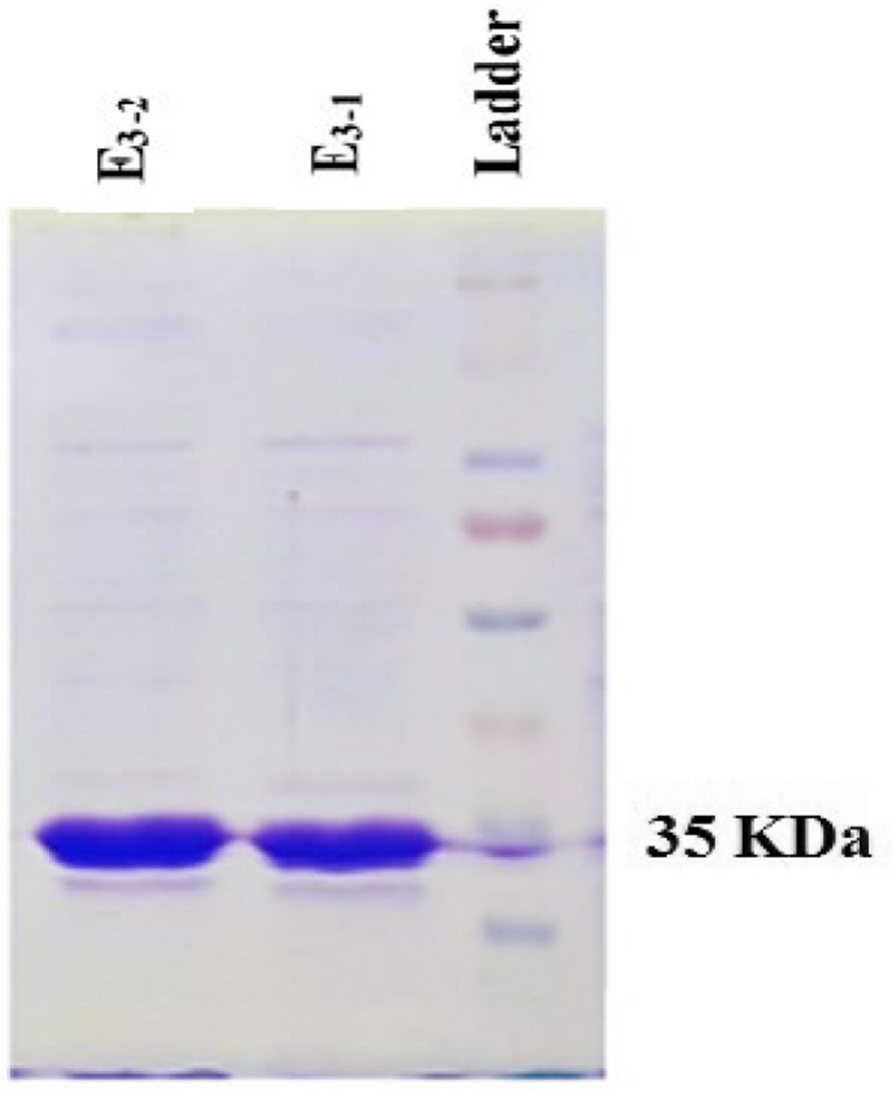
Recombinant urate oxidase purification. proteins eluted collected after Ni-NTA chromatography were visualized on SDS-PAGE.

The recombinant uricase enzyme was purified using previous methods, and the SDS-polyacrylamide gel electrophoresis technique was used to analyze the proteins eluted from the purification [40]. The observation of a single band with a molecular weight in the region of 35 kDa served as conclusive evidence for the expression of recombinant urate oxidase enzyme (Fig. 1) [41].

#### Optimum temperature and incubation time plus taurine concentration by RSM

In order optimize the three most prominent factors (temperatures, incubation time, and the concentration of taurine), a total of 18 full factorial central composite designs (CCDs) were used in this study, using Design-Expert software version 11. The predicted and experimental results are presented in Table 1.

**Table 1:** Design matrix of experiments proposed by response surface methodology (RSM) and the experimental units acquired for each experiment

	Independent variables			Activity (U/ml)	
Run	A: Incubation time (min)	B: Taurine concentration (mmol/L)	C: Temperature (deg C)	Experimental	Predicted
1	25	250	28	1.490	1.490
2	25	250	16	1.780	1.780
3	25	450	28	2.050	2.050
4	15	350	22	1.997	2.000
5	35	150	34	1.447	1.450
6	15	150	22	1.770	1.770
7	25	250	40	1.200	1.200
8	25	250	28	1.490	1.490
9	35	350	34	1.770	1.770
10	25	50	28	1.500	1.500
11	15	350	34	1.800	1.800
12	25	250	28	1.490	1.490
13	5	250	28	1.698	1.700
14	35	350	22	1.450	1.450
15	25	250	28	1.490	1.490
16	35	150	22	1.830	1.830
17	45	250	28	1.728	1.730
18	15	150	34	0.870	0.870

The results shown in Table 1 demonstrate a high degree of concordance between the predicted response values and the corresponding experimental data. The RSM model suggested a quadratic polynomial equation. The ultimate equation, expressed in terms of actual The RSM model suggested a quadratic polynomial equation. The final equation, in terms of the actual factor, can be stated as follows:

$$\mathbf{Activity}=+5.20717-0.049496\,\text{Incubation time}-0.006595\,\text{Taurine concentration}-0.151224\,\text{Temperature}-0.000152\,\text{Incubation time}\,\ast\,\text{Taurine concentration}+0.002154\,\text{Incubation time}\,\ast\,\text{Temperature}+0.000293\,\text{Taurine concentration}\,\ast\,\text{Temperature}+0.000557\,\text{Incubation time}^2+\text{7.12333E-06}\,\text{Taurine concentration}^2-\,\text{4.62963E-07}\,\text{Temperature}^2$$

**Fig. 2:**
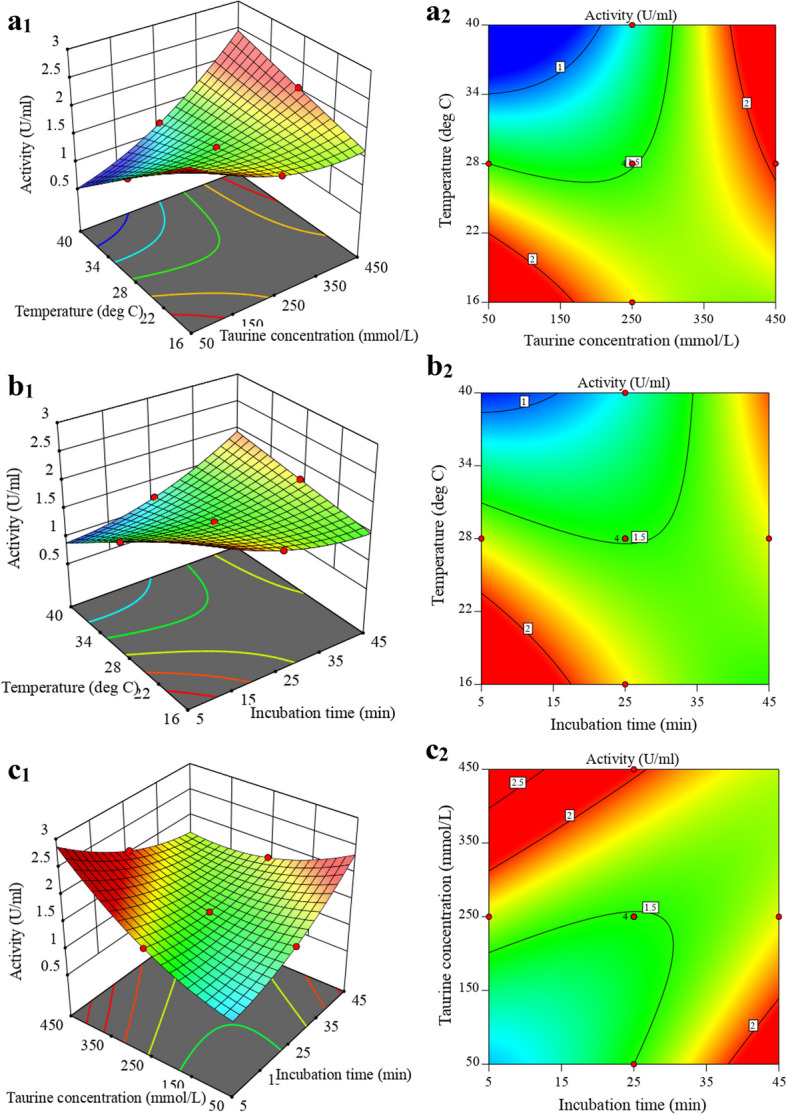
The planned set of contour plots aims to illustrate the impact of three independent variables and their effects on response (activity): (**a1/2**) The influence of temperature, taurine concentration, and their interaction on activity, while keeping the incubation time constant at 25 min (**b1/2**) The impact of temperature, incubation time, and their interaction on activity, while maintaining a constant taurine concentration of 250 mM. (**c1/2**) The effects of taurine concentration, incubation time, and their interaction on activity, while holding the temperature constant at 28 °C

The presence of a positive coefficient indicates a synergistic effect on the response, while a negative coefficient signifies an antagonistic effect. The RSM plot in Fig. 2 illustrates the impact of taurine concentration, incubation time, and temperature on the activity of the recombinant uricase enzyme.

Based on the results shown in Fig. 2a1/2, it can be observed that at high temperatures with increasing taurine osmolyte concentration and at low temperatures with lower taurine osmolyte concentration, the enzyme achieves its highest possible level of activity. Moreover, it is evident from the data presented in Fig. 2b1/2 that the enzymatic activity increases at higher temperatures with increases in the incubation time of treated uricase enzyme with a constant concentration of taurine osmolyte. Moreover, Fig. 2c1/2 indicates that both a low taurine concentration and a long incubation time had a positive effect on the activity of the recombinant uricase enzyme, and a high taurine concentration and a short incubation time had the same effect. These results suggest that the effects of all three variables and their interactions with each other on the recombinant uricase enzyme activity have been significant. The optimum conditions for achieving maximum activity of the recombinant uricase enzyme were determined to be a temperature of 28 °C, a taurine concentration of 450 mM, and an incubation time of 25 min. The recombinant uricase enzyme activity of 2.05 U/ml was generally achieved under optimal conditions, as indicated in Table 1.

### Thermal inactivation and stability

**Fig. 3:**
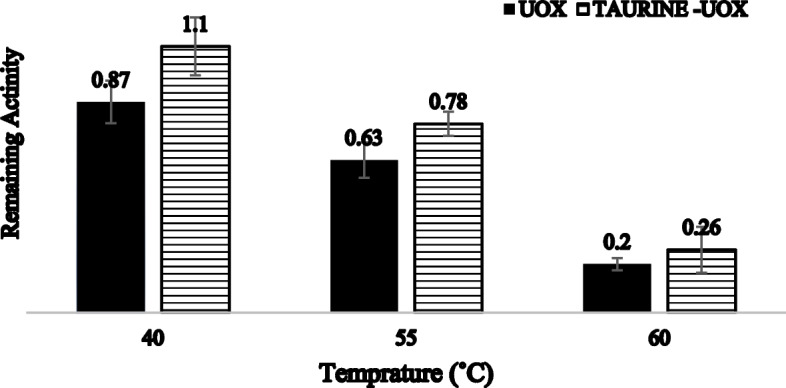
Irreversible thermal inactivation of taurine treated enzyme (white) and pristine enzyme (black) at different temperatures for 60 min

**Fig. 4:**
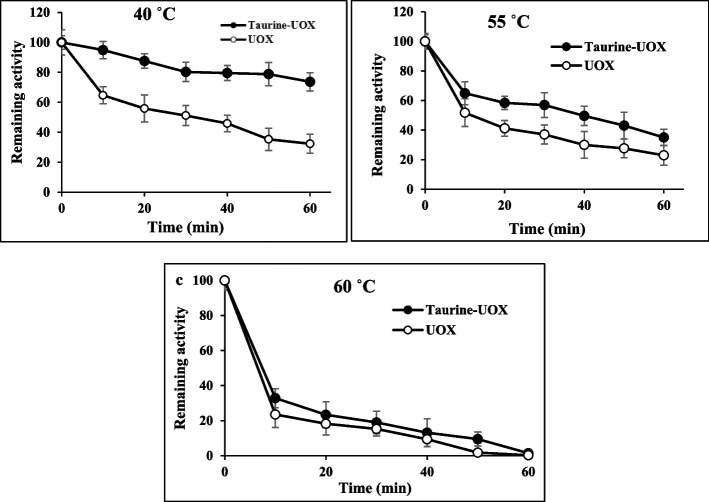
Irreversible thermal inactivation of taurine treated enzyme **a **40 °C, **b** 55 °C, and **c** 60 °C for different time intervals

**Fig. 5:**
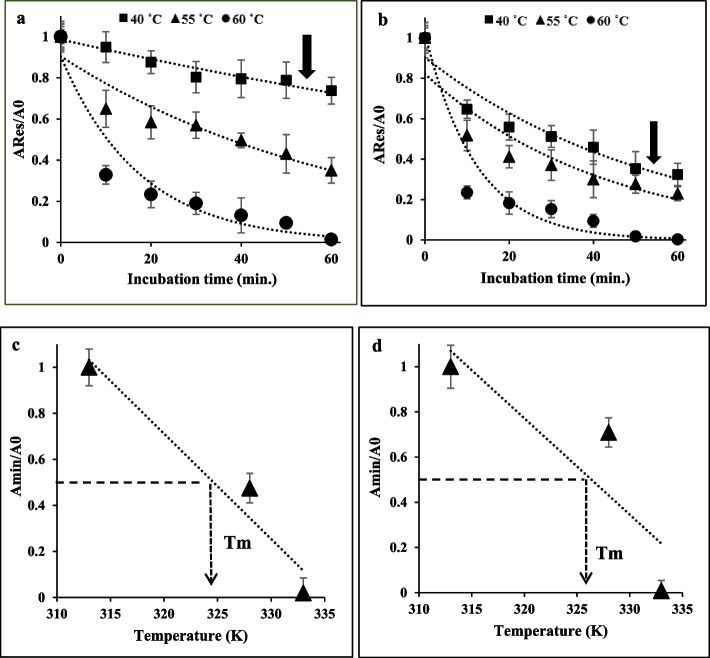
Determination of thermostability parameters: **a** and **b**, Reduction in the proportion of ARes compared with native activity (A0) over time in treated and untreated enzyme. Amin is the minimum activity at a given temperature that corresponds to the enzyme in the equilibrium state which represents the total amount of active enzyme upon cooling **a** the treated enzyme **b** the untreated enzyme. **b** and **c**, Estimation of melting temperature (Tm) that corresponds to the temperature when Amin drops down to 50% of the native activity (A0) in treated and untreated enzymes respectively.

To investigate the mechanism of protein inactivation, it is crucial to assess kinetic and thermodynamic parameters, which are fundamental in thermal processes. The study investigated the irreversible thermal inactivation of enzymes that were treated with taurine and enzymes that were not treated with taurine, at various temperatures. In general, a population of protein molecules undergo irreversible structural changes, which can be seen from the reduction of the residual activity of the pristine enzyme at representative temperatures. Thus, a resetting of the operating temperature does not lead to the reestablishment of the initial enzyme activity. The time and temperature of incubation, as well as the specific protein type, determine the degree of reversibility for each protein. The examination of the thermo-inactivation process following a duration of 60 min indicated that the untreated enzyme exhibited lower stability compared to the enzyme subjected to taurine treatment across the temperature range of 40, 55, and 60 ˚C, particularly at elevated temperatures. The enzyme’s activity was shown to retain 40% and 20% of its initial activity after being incubated for 60 min at 55 °C with and without the presence of taurine, respectively (Fig. 3). After incubating the recombinant uricase enzyme with and without taurine osmolyte at representative temperatures within 40, 55, and 60 ˚C, irreversible thermal inactivation experiments were performed for 1 h for detailed analysis. The results indicated that during a 60 min incubation at 40 ˚C, the enzymes treated with taurine exhibited a retention of over 70% of their activity, as depicted in Fig. 4a, b, and c. The variation in melting points of proteins can be attributed to the distinctive primary sequence of amino acids that are characteristic of certain protein types. Various factors, such as pH and salt concentration, as well as post-translational modifications, can significantly impact the stability of protein structure and hence affect the melting temperature. Protein stability increases proportionally with greater values of the thermal melting temperature (Tm). In order to achieve the minimum level of activity, A_min_, the data of treated and untreated uricase enzyme were plotted until no further drop in enzyme activity (ARes/A0) was observed (Fig. 5a/b). Then, according to Fig. 5c/d, the first parameter of thermal stability, i.e. the melting temperature of the treated and untreated uricase enzyme (T_m_) was estimated.

### Kinetic parameters

**Fig. 6:**
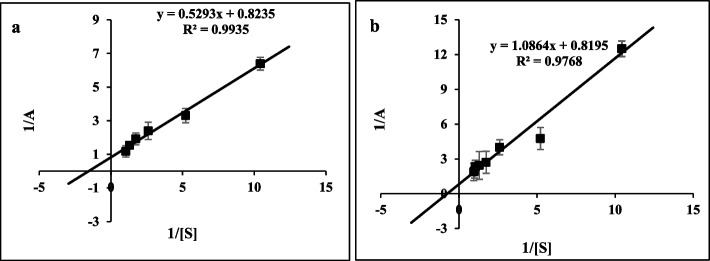
The kinetic parameters of uricase in the absence (**a**) and presence of taurine (**b**)

Based on previous kinetic studies, the Michaelis constant (K_m_) values of the urate oxidase enzyme were investigated under two conditions: in the absence and presence of taurine. Table 2 presents a comprehensive summary of the kinetic parameters acquired in the present study. The taurine-treated enzyme exhibited a twofold augmentation in K_m_ compared to the untreated uricase enzyme. Based on the results, it can be shown that the enzyme treated with taurine osmolyte exhibited a greater affinity towards the substrate in comparison to the untreated uricase enzyme (as depicted in Fig. 6a, b, and Table 2). A high K_m_ value indicates that a substantial amount of substrate is required to achieve enzyme saturation, implying that the enzyme exhibits a low affinity for the substrate. Conversely, a low K_m_ value signifies a minimal quantity of substrate required to achieve enzyme saturation, indicating a higher affinity for the substrate. Hence, the data indicate that there is a change in the rate of conformational change in the presence and absence of taurine on the way to forming the Michaelis complex. An increase in the K_m_ value of the enzyme treated with taurine may indicate that the enzyme’s structure underwent alterations during the stabilization process or that taurine introduces a spatial barrier that hinders the entry of the substrate (uric acid) into the enzyme’s active site [21, 24, 42, 43]. Furthermore, the enzyme’s catalytic efficiency or rate constants for the catalytic conversion of substrate into product (K_cat_) and the ratio of K_cat_ to K_m_, which serves as an indicator of the specificity of the enzyme to the substrate, were examined in both the absence and presence of taurine osmolyte. The observed reduction in the value of this parameter for the enzyme in the presence of taurine osmolyte is probably attributable to the spatial barrier.

**Table 2:** The biochemical characteristics of uricase in the absence and presence of taurine

	V_max_	SD	k_m_	SD	k_cat_	SD	k_cat_/k_m_	SD
UOX	1.210	0.012	0.640	0.015	3.430	0.012	5.350	0.074
Taurine-UOX	1.220	0.021	1.320	0.021	1.010	0.008	0.760	0.006

*SD* standard deviation; *n* = 3

### Fluorescence measurements

#### Fluorescence spectroscopy

**Fig. 7:**
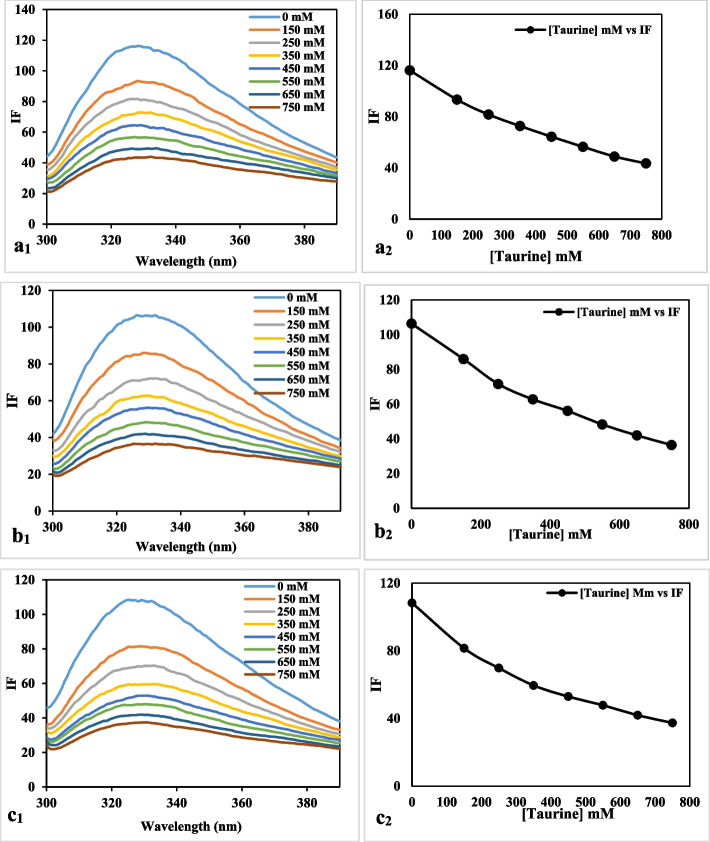
Emission spectra of uricase in the presence of various taurine concentrations of (λ_exc_ = 280 nm) at **a**: 40 ˚C, **b**: 55 ˚C and **c**: 60 ˚C

The utilization of fluorescence spectroscopy has been widely employed to investigate the interactions between ligands and proteins, yielding valuable insights into the quenching mechanism, binding constants, and binding sites [44, 45]. Emission spectra alterations can yield insights about the structure and dynamics of a molecule [46, 47]. The main source of fluorescence in the uricase enzyme is attributed to the presence of tryptophan (Trp) and tyrosine (Tyr) residues. The Trp and Tyr residues of proteins have maximal fluorescence intensities of approximately 340 nm and 300 nm, respectively, when stimulated at 280 nm. Figure 7a, b, and c illustrates the fluorescence spectra of the uricase enzyme at different taurine concentrations. The fluorescence intensity of uricase showed a consistent reduction as the concentration of taurine increased, suggesting that taurine interacted with uricase and influenced its structure [48, 49].

#### Fluorescence quenching mechanism

Fluorescence quenching refers to the reduction in the efficiency of fluorescence emission by a fluorophore due to various chemical interactions, including excited-state reactions, formation of ground-state complexes, energy transfer, and collisional quenching. The methods of quenching can be categorized as either dynamic quenching or static quenching. These categories are differentiated by their respective dependencies on temperature and viscosity, or preferable by measuring their lifetimes. As the temperature increases, the diffusion coefficients also increase. Consequently, the dynamic quenching constants will also increase with rising temperature. In contrast, the rise in temperature is expected to lead to a reduction in the stability of complexes. Therefore, it is anticipated that the values of the static quenching constants will be lower [50]. The well-known Stern–Volmer equation was used to confirm the mechanism as follows [51]:

$$\frac{{F}_{0}}{F}=1+{K}_{q}{{\uptau }}_{0}\left[Q\right]=1+{K}_{SV}\left[Q\right]$$

F_0_ and F denote the fluorescence intensity when there is no quencher and when the quencher is present, respectively; [52] represents the concentration of the quencher, while τ_0_ denotes the fluorescence lifetime when there is no quencher present. The value of τ_0_ is always 10^−8^ seconds [53]. K_q_ represents the rate at which the biological macromolecule is quenched, whereas K_SV_ denotes the Stern-Volmer quenching constant. K_SV_ is also denoted by the following equation:

$${K}_{SV}={K}_{q}{{\uptau }}_{0}$$

**Fig. 8:**
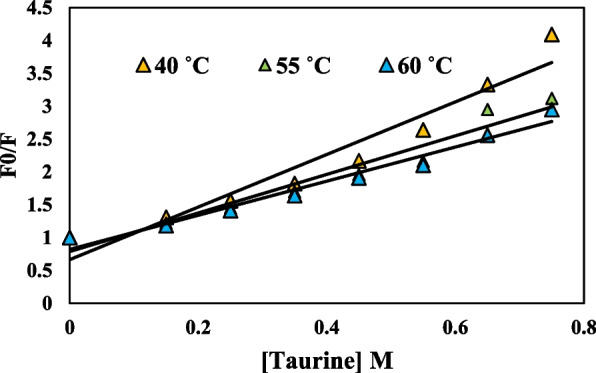
Stern–Volmer plots for the quenching of uricase by taurine at 40, 55, and 60 ˚C

This study aimed to investigate the quenching mechanism of uricase by obtaining fluorescence quenching spectra at three distinct temperatures (40, 55, and 60 °C) in the presence of various taurine concentrations. Figure 8 displays the Stern-Volmer plots depicting the quenching of uricase fluorescence by taurine. The computed K_SV_ and K_q_ values are presented in Table 3. A linear Stern-Volmer plot typically suggests the presence of a single class of fluorophore, which is equally susceptible to quenching by the quencher. The assessment of static and dynamic quenching can be determined by observing the impact of temperature and viscosity. Another, more preferable approach is to conduct studies on fluorescence lifetime [22]. Increasing temperatures cause diffusion to occur more quickly, leading to an increase in collisional quenching. Additionally, higher temperatures typically cause loosely bound complexes to separate, resulting in a decrease in static quenching. Dynamic quenching is indicated by the rise in K_SV_ value with increasing temperature. The K_q_ value was < 2.0 × 10^10^ L mol^−1^ s^−1^. Furthermore, the observation suggests that the decrease in uricase fluorescence caused by taurine is a form of static quenching, as seen in Fig. 7a, b, and c. The impact of dynamic quenching is widely acknowledged to affect only the excited state of the fluorophore, whereas static quenching alters the absorption spectrum of the fluorophore. This study showed that the quenching process occurs through static quenching, which is triggered by the formation of the uricase-taurine complex in its ground state. Osmolytes tend to increase the stability of proteins’ natural structure. In conclusion, these findings suggest that the interaction between the protein and the quencher is a complex one, which is more consistent with the static quenching process rather than dynamic collision quenching.

**Table 3:** Stern-Volmer constants for the interaction of uricase with taurine at different temperatures

T (K)	K_SV_ × 10^−2^ (L mol^−1^)	K_q_ (10^9^ M^−1^ s ^−1^)	R^2^
313	**400.070**	**0.400**	**0.954**
328	**293.410**	**0.293**	**0.975**
333	**259.560**	**0.259**	**0.985**

#### Calculation of binding parameters

**Fig. 9:**
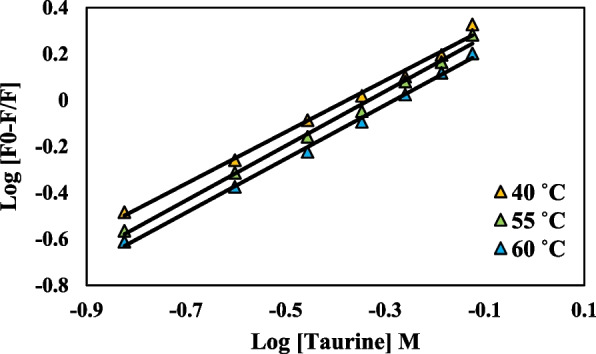
Double-log plots of the taurine quenching effect on uricase enzyme fluorescence at different temperatures

The equation provided can be used to compute the binding constant (K_b_) and the number of binding sites (n) in the static quenching process, where small molecules independently bind to a group of identical sites on a macromolecule.

$$\text{log}\left(\frac{{F}_{0}-F}{F}\right)=log{K}_{a}+nlog\left[Q\right]$$

where [52] is the concentration of the quencher; F_0_ and F are the fluorescence intensity in the absence and presence of the quencher, respectively; K_a_ is the binding constant, and n is the number of binding sites per uricase molecule. By plotting the logarithm of (F_0_ − F)/F against the logarithm of [52] (as depicted in Fig. 9), one may determine the values of n and K_a_ [54]. The results are succinctly presented in Table 4.

Significantly, the values of n at the experimental temperatures were nearly identical to 1, suggesting that there is just one binding site in uricase for taurine. Furthermore, the increase in temperature resulted in a decrease in the K_a_ value, indicating a decrease in the stability of the uricase-taurine complex. Therefore, the binding process was characterized as an exothermic reaction.

**Table 4:** Binging and thermodynamic parameters of UOX-Taurine interaction at three different temperatures

T (K)	K_a_ × 10^−2^ (L mol^−1^)	n	ΔH (kJ mol^−1^)	ΔG (kJ mol^−1^)	ΔS (J mol^−1^ K^−1^)
313	**262.970**	**1.110**	**-77.750**	**-25.530**	**-16.680**
328	**246.890**	**1.170**		**-23.020**	
333	**213.060**	**1.160**		**-22.190**	

##### Thermodynamic parameters determination

**Fig. 10:**
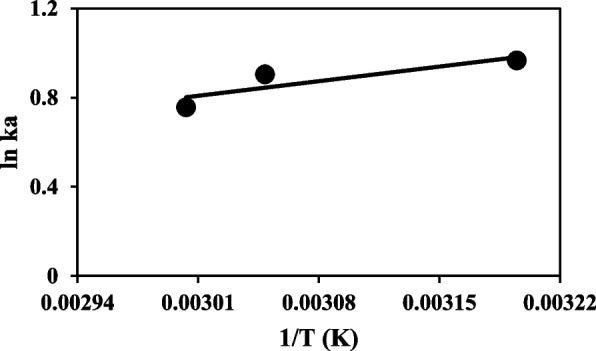
plot lnK_a_ against 1/T for interaction taurine with uricase at 313, 328, and 333 K

The binding constant is influenced by temperature, indicating that the thermodynamic process is responsible for the creation of the uricase-taurine complex. An analysis of this dependency was made to reveal the interacting forces between taurine and uricase. Four non-covalent-type interactions attribute a major part of the bonding between ligands to themselves. The hydrophobic force, van der Waals forces, hydrogen bonding, and electrostatic forces are some of these interactions [22]. The major forces were determined based on the sign and magnitude of the thermodynamic parameters. The Vant Hoff equation can be used to approximate the value of ΔH and ΔS if they are only slightly variable and do not change much over the studied temperature range.

$$\begin{array}{ll}Ln{K}_{a}=-\frac{\varDelta H}{RT}+\frac{\varDelta S}{R}\\\varDelta G=-RTLn{k}_{a}=\varDelta H-T\varDelta S\end{array}$$

where K_a_ and T refer to the binding constant and temperature, respectively, and R is the gas constant (8.314 J mol^−1^ K^−1^). The ΔG values were determined and the obtained results were shown in Table 4. Also, the ΔH and ΔS values were calculated by graphing $Ln{k}_{a}$ vs. $\frac{1}{T}$ (Fig. 10).

Ross and Subramanian have provided a comprehensive summary of the forces that govern the interactions between proteins and ligands, allowing for the identification of many forms of binding with distinct interactions. If the enthalpy change (ΔH) is negative and the entropy change (ΔS) is negative, then the binding reaction is mostly governed by van der Waals forces and hydrogen bond interactions. When the change in enthalpy (ΔH) is greater than zero and the change in entropy (ΔS) is also greater than zero, it indicates that hydrophobic interactions are the prevailing factor. If the change in enthalpy (ΔH) is negative and the change in entropy (ΔS) is positive, then the primary driving factors are electrostatic interactions. Enthalpy change (ΔH) can be considered constant when the temperature range is minimal. The utilization of enthalpy change (ΔH) and entropy change (ΔS) can serve to validate the binding modalities [55]. The interaction and forces governing the relationship between taurine and uricase can be described as follows: The negative values of ΔS and ΔH indicate that the van der Waals force and the hydrogen bonding interactions were the main factors influencing in the interaction of taurine with UOX. Furthermore, the negative ΔG values suggest that the binding process occurred spontaneously.

#### ANS fluorescence

**Fig. 11:**
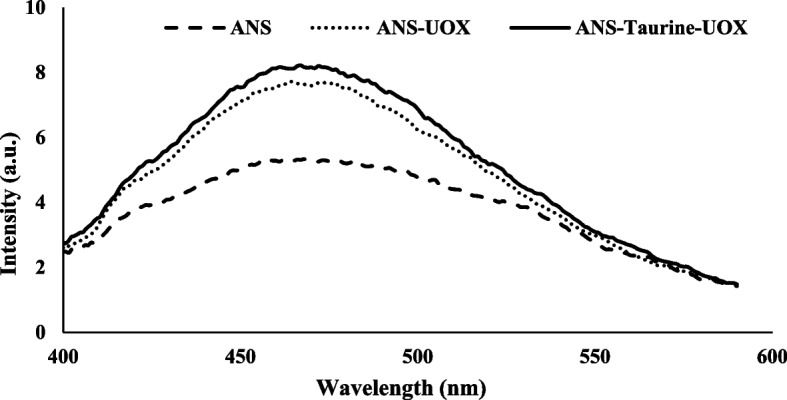
Fluorescence study of uricase in the absence and presence of taurine. Extrinsic fluorescence measurement of uricase using ANS. ANS (dashed line), ANS along with free uricase (dotted line), along with taurine treated uricase (solid line); The spectrums were taken by excitation at 280 nm

The fluorescent probe ANS is employed for the purpose of identifying protein binding sites. The present investigation involved the utilization of spectroscopy techniques under controlled conditions, specifically at a temperature of 28 °C. The excitation wavelength employed was 370 nm, whereas the emission wavelength range spanned from 400 to 600 nm. Figure 11 displays the fluorescence emission curves pertaining to the interaction between ANS and both treated and untreated uricase enzymes. After the binding of ANS to the hydrophobic surface of the treated enzyme, there was a significant increase in fluorescence intensity. The results suggest that there has been a modification in the composition and conformation of the enzyme’s tertiary and quaternary structures, which has led to the placement of hydrophobic residues on the enzyme surface. This may allow for the establishment and arrangement of more stable hydrophobic regions or clefts by increasingly shielding them from a polar environment, thereby driving equilibrium towards the formation of a more stable protein complex [23, 56–60].

### Molecular dynamics simulation results

MD simulations can be a valuable mean to gaining insight into the conformational properties of the enzyme [40, 61]. Our obtained theoretical results are valuable to improve the stability and performance development of UOX. MD simulations for native and taurine-treated enzymes were done through MD simulation package GROMACS 5.1.4 which adopts the CHARRM27 force field parameter for energy. To understand the enzyme activity, it is necessary to examine the protein structure in the presence of taurine.

#### RMSD and secondary structure analysis

**Fig. 12:**
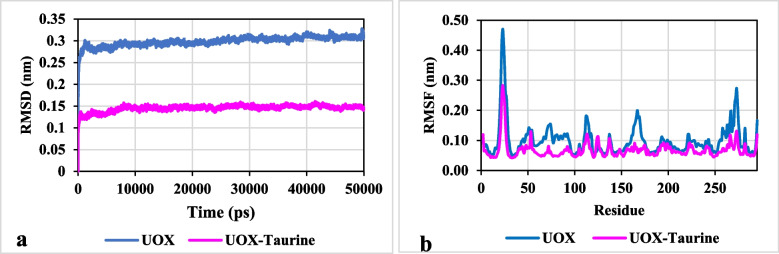
The root mean square deviation of Cα atoms (Cα-RMSD) and the root mean square fluctuation of Cα atoms (Cα-RMSF) of uricase in the absence (blue line) and presence of taurine (pink line)

The root mean square deviation (RMSD) and the root mean square fluctuation (RMSF) of Cα atoms of two simulated enzymes were determined and illustrated in Fig. 12a and b.

The RMSD values reached saturation very fast, after about 2.3 ns, after which the variations reached the minimum values (see Fig. 12a). This evidence suggests that each system is simulated for a sufficient time to reach equilibrium and that the simulation time is enough to cover all possible states. At the end of the simulation, the RMSD values of naked and treated enzymes reached ~ 0.310 nm and ~ 0.144 nm, respectively. The lower Cα-RMSD value of the enzyme in the presence of taurine compared to the free enzyme indicates the stability of the UOX structure in the binary system.

The structural flexibility of both the naked and taurine-treated enzymes was assessed by calculating the Cα-RMSF (refer to Fig. 12b). In general, the pure water system exhibited greater fluctuations in residues compared to the binary system. Regions with higher flexibility were characterized by higher RMSF values. Consequently, the lower RMSF values observed in the binary system confirm that treating UOX with taurine enhances the stability of the enzyme.

**Fig. 13:**
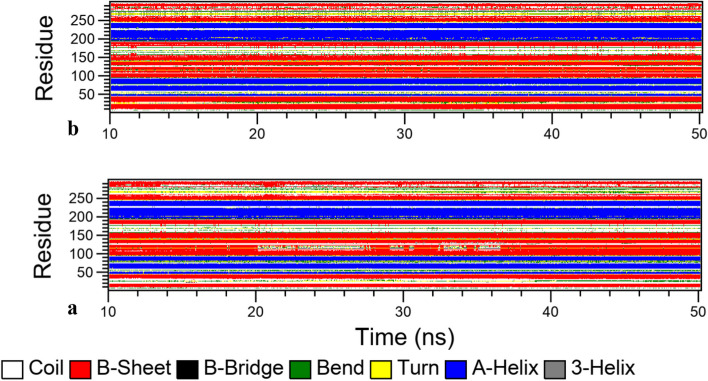
Secondary structure assignment of the protein as a function of time for **a**: UOX and **b**: UOX/taurine for 40 ns

The information about the local secondary structure changes has been obtained from the MD simulation. The UOX secondary structure during the 40 ns of MD simulations was investigated to examine the changes in the content of α-helix and β-sheet structures in the enzyme (See Fig. 13a and b).

According to these results, the higher β-sheet and α-helix content of taurine-treated enzyme suggest that taurine may be responsible for the stability and higher activity of the enzyme at elevated temperatures and may preserve the enzyme’s active site integrity.

Table 5 reports the percentage of the secondary structures for the free and treated enzymes during the MD simulation.

**Table 5:** Secondary structure of UOX in the two simulated systems

	Coil	β -Sheet	β-Bridge	Bend	Turn	α-Helix	3-Helix
UOX	25.75%	32.61%	1.13%	9.72%	5.64%	23.90%	0.57%
UOX-Taurine	21.30%	37.46%	1.21%	6.38%	6.84%	25.12%	1.01%

The α-helix and β-sheets contents of the taurine-UOX enzyme (25.12% and 37.46%, respectively) increased compared with those of the free enzyme (23.90% and 32.61%, respectively). Further, the obtained results indicate that the β-bridge percentage in the treated enzyme has been higher than that of the naked enzyme (see Table 5). The obtained MD simulation results provide more details about the taurine effects on the enzyme structure.

The enzyme stability is estimated by a number of interactions including hydrogen bonding, electrostatic, hydrophobic interactions, and van der Waals forces [62, 63]. Thus, the taurine-UOX interaction changes the secondary structure composition of the enzyme. The hydrogen bond between the carbonyl oxygen and the amide hydrogen atoms forms the α-helix and β-sheet structures; thus, changes in the secondary structure composition of UOX may enhances its intramolecular H-bonds so that the content of the α-helix and β-sheet increases. In general, the results showed that taurine causes changes in the composition of the enzyme and thus boosts the enzyme activity, which is consistent with the results of the experimental section. The research findings confirmed the significant impact of taurine on the UOX stability.

**Fig. 14:**
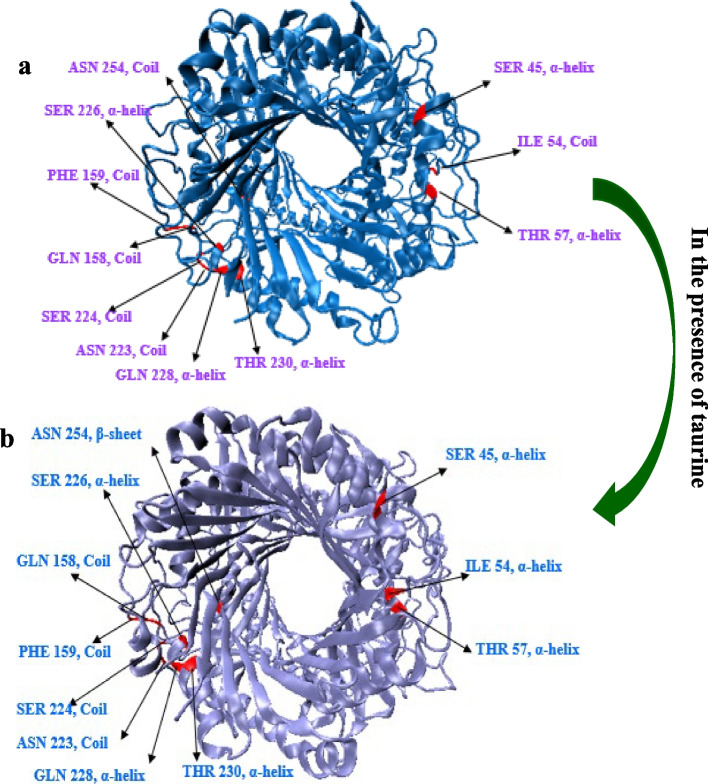
The structures of a: free UOX and b: treated-taurine UOX at the end of 50 ns simulation (active site residues have been indicated with red color)

In Fig. 14, it can be observed that the enzyme’s conformation was changed from a random coil to a β-sheet and α-helix in the binary system. This change in conformation could potentially maintain the integrity of the enzyme’s active site, contributing to its increased activity and stability at elevated temperatures.

#### SASA and hydrogen bonding

**Fig. 15:**
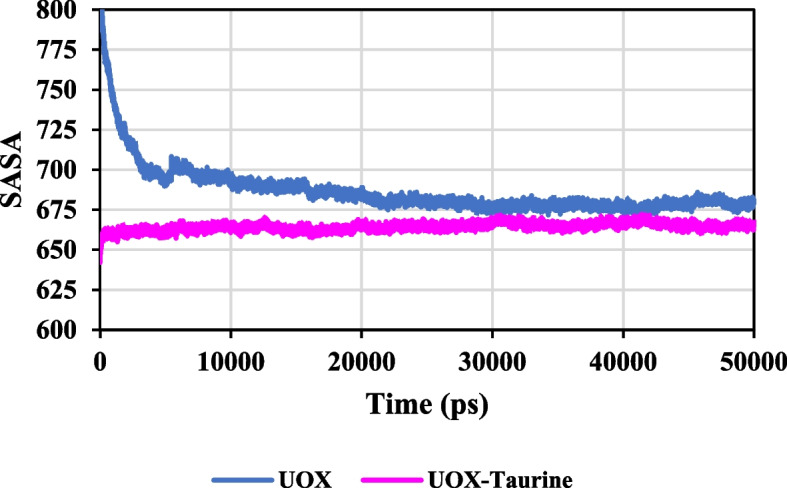
The SASA value of enzyme in the presence and absence of taurine

Solvent accessible surface area (SASA) is a significant parameter to analyze the interactions between protein and solvent [64]. In other words, the SASA values show the conformational stability of the enzyme [65, 66]. The SASA values for the 50 ns simulation trajectory have been plotted in Fig. 15.

It is shown in Fig. 15 that the SASA value of UOX-taurine has diminished, which indicates that in the presence of taurine, the intramolecular hydrogen bonding of UOX grows, thus its accessible surface area by solvent molecules drops. The lower SASA value shows the higher thermodynamic stability of the protein. Thus, it can be stated that the lower SASA value in the UOX-taurine system shows a more compact enzyme structure as well as higher thermodynamic stability of UOX in the binary system (see Fig. 15).

**Fig. 16:**
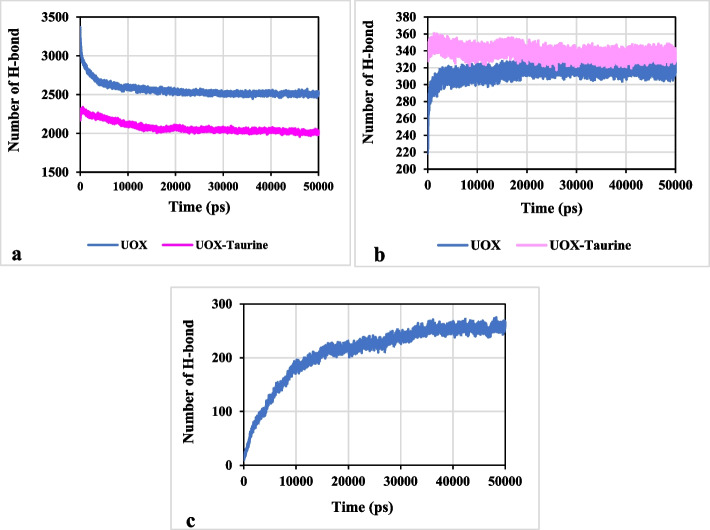
The number of hydrogen bonds **a**: between UOX and water molecules and **b**: intra-protein and **c**: between UOX and taurine molecules

Figure 16a reveals that the number of H-bonds between enzyme and water molecules in the UOX-taurine system has been lower than that of the UOX system, which is related to the enhancement in the number of intra-protein hydrogen bonds in the binary system (see Fig. 16b). Comparison of Figs. 15 and 16a shows that the SASA value corresponds well with the number of H-bonds between the UOX enzyme and water molecules.

The taurine molecule, due to having hydrogen and hydroxyl oxygen atoms, can participate in the formation of hydrogen bonds with the UOX side chains (Fig. 16c). Thus, another reason for reduced number of H-bonds between UOX and water molecules may be related to the formation of hydrogen bonds between the enzyme and taurine molecules. In general, these results imply that the main driving forces for stabilizing enzyme structure are sharing hydrogen bonds between enzyme- enzyme and enzyme- taurine.

### Molecular docking results

Molecular docking is a method used to predict the binding behavior and affinity between specific molecules and their template proteins in noncovalent binding [67, 68]. The target-based approach like docking can only be applied to proteins whose 3D structures have been identified [52]. In this study, docking simulations were conducted to analyze the interaction between taurine and the active site of the UOX enzyme. A total of 100 different conformations of taurine were examined during the docking process, and the conformation with the lowest binding energy was selected for further analysis. The results, shown in Table 6, include information on hydrogen bond and van der Waals energy, approximated free energy of binding, final intermolecular energy, final total internal energy, electrostatic energy, estimated inhibition constant, and torsional free energy.

**Table 6:** Docking results with the interacting residues

Lowest binding energy (kcal/mol)	vdW + Hbond + desolv energy (kcal/mol)	Torsional free energy (kcal/mol)	
**-4.400**	**-3.940**	**1.190**	
Inhibition constant (µM)	Electrostatic energy (kcal/mol)	**Interaction bond**	
		Hydrogen-bonding	Hydrophobic-bonding
**598.780**	**-1.650**	**ASN100**	**Thr33**
Final intermolecular energy (kcal/mol)	Final total internal energy (kcal/mol)	**ASN12**	**Val34**
**-5.590**	**-2.520**	**ASP11**	**Cys35**

**Fig. 17:**
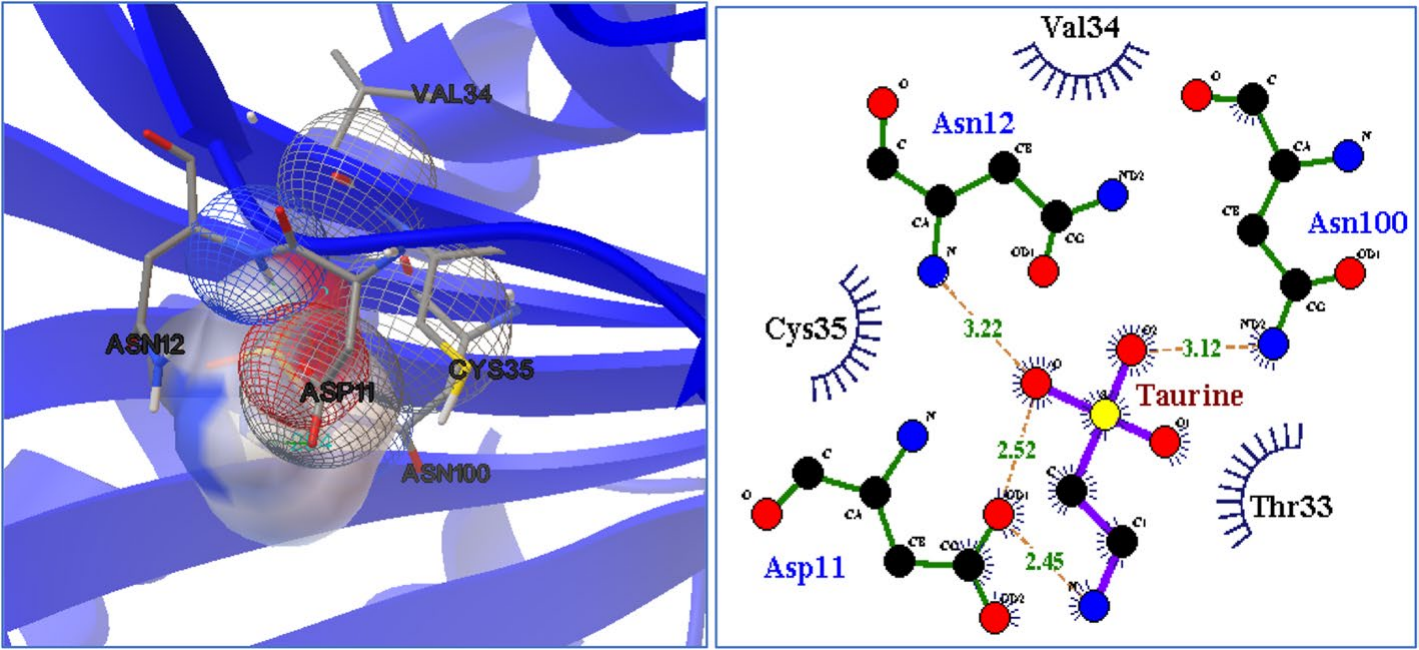
Molecular docking analysis results. Docking simulation shows that A: Taurine molecule is located within the hydrophobic cavity of UOX active site. The orange dotted lines indicate the formation of the hydrogen bond

The presence of polar and hydrophobic residues around taurine suggests that the binding process is primarily driven by electrostatic and hydrophobic interactions. The negative value of the free binding energy confirms that UOX automatically binds to taurine. The best-docked orientation of taurine with the UOX active site was illustrated in Fig. 17.

Additionally, the molecular docking data predicted the formation of hydrogen bonds between taurine and the binding pocket (Fig. 17). Therefore, these docking results confirmed the observations from the quenching fluorescence. Additionally, the quenching fluorescence findings suggested that the hydrogen bonding network and van der Waals forces were the primary binding forces contributing to the UOX-taurine interaction.

## Conclusion

The obtained results suggest that estimating the optimal conditions to obtain the maximum activity using RSM-based CCD can save cost and time. The best conditions for UOX activity were obtained at a temperature of 28 °C, a taurine concentration of 450 mM, and an incubation time of 25 min. In general, the uricase activity of 2.05 U/ml was obtained under optimal conditions. The investigation of kinetic and thermodynamic parameters was conducted to study the mechanism of protein inactivation, which is an essential parameter in thermal processes. The results of the experiment indicated that the untreated urate oxidase enzyme exhibited lower stability under elevated temperatures in comparison to the enzyme treated with taurine. The K_m_ values for the urate oxidase enzyme were investigated in the presence and absence of taurine osmolyte, and a two-fold increase in K_m_ for the taurine-treated enzyme was observed, suggesting that it had a lower affinity for the substrate compared to the untreated enzyme. The other word, a low K_m_ value signifies a minimal quantity of substrate required to achieve enzyme saturation, indicating a higher affinity for the substrate. Furthermore, MD simulation results showed that changes in the secondary structure composition of uricase enzyme enhanced its intramolecular hydrogen bonds, increasing the contents of α-helix and β-sheet. The presence of hydrogen bonds between the enzyme and taurine molecules also decreased the dynamics of water molecules, which had a significant role in the protection of protein structure. The ANS fluorescence of the enzyme was investigated, and the increased fluorescence intensity of the taurine-uricase-ANS complex compared to uricase-ANS indicated greater stabilization of the enzyme structure in the presence of taurine. Finally, the molecular docking results with the negative free binding energy value confirm that UOX automatically binds to taurine.

### Supplementary Information

**Additional file 1: Figure S1.** The root mean square deviation of Cα atoms (Cα-RMSD) of uricase in the absence (A) and presence of taurine (B) during the simulation (first run, second run and third run). **Figure S2.** The SASA value of uricase in the absence (A) and presence of taurine (B) during the simulation (first run, second run and third run). **Figure S3.** The number of hydrogen bonds between water molecules and uricase in the absence (A) and presence of taurine (B) during the simulation (first run, second run and third run). **Figure S4.** The number of hydrogen bonds within the protein in the absence (A) and presence of taurine (B) during the simulation (first run, second run and third run). **Figure S5.** The number of hydrogen bonds between UOX and taurine molecules during the simulation (first run, second run and third run).

## Data Availability

Not applicable.

## Abbreviations

UOX: urate oxidase
CCD: central composite designs
MD: Molecular dynamics
RMSD: Root-mean-square deviation
SASA: Solvent-accessible surface area
