# Supplementary material for: Exploring the impact of taurine on the biochemical properties of urate oxidase: response surface methodology and molecular dynamics simulation
Source: J Biol Eng. 2024 Jan 22;18:10. doi: 10.1186/s13036-023-00397-x (PMC10804793; doi:10.1186/s13036-023-00397-x)
Supplement: Supplementary file 1 — Additional file 1: Figure S1. The root mean square deviation of Cα atoms (Cα-RMSD) of uricase in the absence (A) and presence of taurine (B) during the simulation (first run, second run and third run). Figure S2. The SASA value of uricase in the absence (A) and presence of taurine (B) during the simulation (first run, second run and third run). Figure S3. The number of hydrogen bonds between water molecules and uricase in the absence (A) and presence of taurine (B) during the simulation (first run, second run and third run). Figure S4. The number of hydrogen bonds within the protein in the absence (A) and presence of taurine (B) during the simulation (first run, second run and third run). Figure S5. The number of hydrogen bonds between UOX and taurine molecules during the simulation (first run, second run and third run). [file 13036_2023_397_MOESM1_ESM.docx]

**A**

**Figure S1.** The root mean square deviation of Cα atoms (Cα-RMSD) of uricase in the absence (A) and presence of taurine (B) during the simulation (first run, second run and third run).

**Figure S2.** The SASA value of uricase in the absence (A) and presence of taurine (B) during the simulation (first run, second run and third run).

**A**

**Figure S3.** The number of hydrogen bonds between water molecules and uricase in the absence (A) and presence of taurine (B) during the simulation (first run, second run and third run).

**B**

**Figure S4.** The number of hydrogen bonds within the protein in the absence (A) and presence of taurine (B) during the simulation (first run, second run and third run).

**Figure S5.** The number of hydrogen bonds between UOX and taurine molecules during the simulation (first run, second run and third run).
